# Legume Lectin FRIL Preserves Neural Progenitor Cells in Suspension Culture In Vitro

**DOI:** 10.1155/2008/531317

**Published:** 2008-08-05

**Authors:** Hailei Yao, Xiaoyan Xie, Yanhua Li, Dongmei Wang, Shu Han, Shuangshuang Shi, Xue Nan, Cixian Bai, Yunfang Wang, Xuetao Pei

**Affiliations:** Stem Cells and Regenerative Medicine Lab, Beijing Institute of Transfusion Medicine, Beijing 100850, China

## Abstract

In vitro maintenance of stem cells is crucial for many clinical applications. Stem cell preservation factor FRIL (Flt3 receptor-interacting lectin) is a plant lectin extracted from *Dolichos Lablab* and has been found preserve hematopoietic stem cells in vitro for a month in our previous studies. To investigate whether FRIL can preserve neural progenitor cells (NPCs), it was supplemented into serum-free suspension culture media. FRIL made NPC grow slowly, induced cell adhesion, and delayed neurospheres formation. However, FRIL did not initiate NPC differentiation according to immunofluorescence and semiquantitive RT-PCR results. In conclusion, FRIL could also preserve neural progenitor cells in vitro by inhibiting both cell proliferation and differentiation.

## 1. INTRODUCTION

Stem cells are unique cells that retain the ability to divide and proliferate throughout postnatal life to provide progenitor cells that can progressively become committed to produce specialized cells. Many cytokines have been reported as inducers of stem cell proliferation and differentiation, one of them is FL (Flt3 ligand) which can prolong maintenance of primitive human cord blood cells in stromal-free suspension cultures [1–4]. However, FL-containing cultures still cause the loss of repopulating capacity of primitive cells [5–10]. 

We have extracted and identified a new legume lectin from Hyactinth bean [11]. It is a ligand of receptor tyrosine kinase Flt3 which is strictly expressed in hematopoietic and neural cell lines [12–14]. Gabriella has reported a new flt3 ligand which has the ability to preserve hematopoietic progenitor cells in vitro for 4 weeks [15]. The gene and protein sequences of our protein are quite similar to Gabriella’s protein [11], besides our protein is also capable of long time in vitro preservation of hematopoietic progenitor cells. According to previous reports [15–19], we named our protein FRIL too and found that FRIL could maintain hematopoietic stem cell in *G*_0_/*G*_1_ phase for at least 4 weeks. Low activation of FRIL’s receptor Flt3 is considered the key mechanism through which FRIL preserves cells. Further experiments showed that FRIL inhibited hematopoietic stem/progenitor cell (HSPC) apoptosis by upregulating p53, reduced activation of MAPK and phosphorylation of STAT5, disturbed formation of AP-1 complex, and in the end suppressed the expression/activation of cell cycle proteins [20–22].

Over the last decade, neural stem cell research provided penetrating insights into plasticity and regenerative medicine. Stem cells have been isolated from both embryonic and adult nervous system [23, 24]. Although with the help of extrinsic signaling factors progress has been made in understanding fundamental stem cell properties, processes that determine cell fate or that maintain a cell’s primitive state are still unknown. As a following experiment to find out whether FRIL can preserve neural progenitor cells as it does on HSPCs, we first identified the isolated neural progenitor cells (NPCs) and their flt3 expressing levels, then we supplemented FRIL into different NPC suspension culture media, observed cell morphological changes, analyzed cell growth and cell surface marker expression, finally we used semiquantitive RT-PCR to verify the changes in gene expression.

## 2. MATERIALS AND METHODS

### 2.1. NPCs isolation and cell culture

E13.5 SD rats (experimental animals department, Peking University, Beijing, China) were sacrificed, then fetal embryonic telencephalon and mesencephalon were dissected under the dissecting microscope, after digestion in the sterilized tube, cells were seeded in 25 cm^2^ flask for general culture and passage. Meanwhile statistically supplemented EGF(20 ng/mL), bFGF(20 ng/mL), and FRIL(300 *μ*g/mL, 1 : 1000) into basic culture media(98% DMEM/F12 (1 : 1) and 2% B27/N2) to form eight groups of culture media (control, EGF, bFGF, FRIL, EGF&bFGF, EGF&FRIL, bFGF&FRIL, EGF&bFGF&FRIL). After second passage, equal numbers of NPCs were seeded with these different culture media. Furthermore, some NPCs were seeded in differentiation media which contains 5% fetal bovine serum. Culture media comprised a low percent of glucose and did not contain mannose which may exerted negative effect on FRIL’s function [15].

### 2.2. Identification of NPCs and expression of flt3 on NPCs

P2 neurospheres were transferred into poly-l-lysine (pll) precoated 24-well plate, incubated for 8 hours at 37°C, and then stained by nestin for immunofluorescence for the purpose of NPCs identification. Meanwhile, some other neurospheres were treated with HRP conjugated FRIL in order to find out the proportion of flt3 expressed on NPCs.

### 2.3. Cell counting assay

Cells were harvested at day 7 following seeding, resuspended with PBS after digestion and centrifuge, and then seeded on 96-well plate together with 100 *μ*L solution and 10 *μ*L CCK-8(Cat. no. CK04, Dojindo Laboratories Co, Japan.) per well. Plate was incubated at 37°C, for 4 hours, then the absorbance at 450 nm was measured using microplate reader with a reference wavelength at 650 nm(Bio-Rad Model 550). Data was analyzed by SAS 8.2 software.

### 2.4. Morphological observation

P3 NPCs were harvested and digested into single cell, cells were counted and seeded into 25 cm^2^ flasks with a desity of 5 × 10^4^ per mL. Each flask was supplemented with only one media described above, and NPC morphology was observed everyday.

### 2.5. Immunofluorescence assay

NPCs were seeded into pll precoated 24-well plate and incubated at 37°C for 8 hours, fixed with 4% paraformaldehyde for 20 minutes, washed with PBS 5 minutes three times, 0.3% Triton X-100 20 minutes, PBS 5 minutes three times, then were incubated with primary antibody for 2 hours at 37°C, washed, and incubated with secondary antibody for 30 minutes at 37°C. Primary antibodies that were used include nestin (Mouse Anti-Nestin Monoclonal Antibody, Cat. no. MAB353, Chemicon, Inc, USA.), GFAP (Cat. no. ZA-0117, Beijing Zhongshan Golden Bridge Biotechnology Co., Ltd.), NSE (RABBIT ANTI-NSE, Cat. no. BA0535, Boster Biotechnology Co. Ltd.), Tubulin (Monoclonal Anti-*β*-Tubulin III antibody produced in mouse Clone SDL.3D10, ascites fluid, Cat. no. T 8660, Sigma-Aldrich Co, USA.), O4 (Mouse Anti-Oligodendrocyte Marker O4 Monoclonal Antibody, Cat. no. MAB345, Chemicon, Inc. USA.). All primary antibodies were diluted with PBS and 10% normal goat serum. Secondary antibodies included FITC (Fluorescein-Conjugated) AffiniPure Goat Anti-Mouse IgG (H+L) (Cat. no. ZF-0312, Beijing Zhongshan Golden Bridge Biotechnology Co., Ltd.), Rhodamine (TRITC)-Conjugated AffiniPure Goat Anti-Mouse IgG (H+L) (Cat. no. ZF-0313, Beijing Zhongshan Golden Bridge Biotechnology Co., Ltd.), Rhodamine (TRITC)-conjugated Affinipure Goat Anti-Rabbit IgG (H+L) (Cat. no. ZF-0316, Beijing Zhongshan Golden Bridge Biotechnology Co., Ltd.). When cells were stained for nestin together with GFAP, Tubulin or NSE, primary and secondary antibodies for each were added simultaneously. 4′,6′-Diamidino-2-phenylindole dihydrochloride (DAPI; 1 : 10000) was used in all fluorescence facilitating cell counts. Images for cell counts were captured from six equally spaced, predetermined areas of each well under fluorescence microscope. NPCs, neurons, glials, and other cells on the images were counted and analyzed.

### 2.6. Semiquantitive RT-PCR assay

mRNA was isolated using trizol kit according to manufacture protocols, then mRNA was reverse transcripted in a final volume of 100 *μ*L, 42°C 5 minutes, 42°C 1 hour, 94°C 5 minutes, −20°C. Then, *ACTIN* F 5′TACCACTGGCATCG RGATGGACT 3′ R 5′TCCTTCTGCATCCTGTCGGCAAT 3′ 94°C 30 seconds, 59°C 30 seconds, 72°C 30 seconds, 25 cycles, 72°C 5 minutes, 4°C. *GFAP* F 5′GAAGAAAACCGCATCACCAT3′ R 5′GCACACCTCACATCACATCC3′ 94°C 30 seconds, 57°C 30 seconds, 72°C 30 seconds, 25 cycles, 72°C 5 minutes, 4°C. *Nestin* F 5′GGAGCCATTGTGGTCTACTGA3′ R 5′TCCCACCGCTGTTGATTT3′ 94°C 30sec, 56°C 30 seconds, 72°C 30 seconds, 28 cycles, 72°C 5 minutes, 4°C. *NeuroD1* F 5′ACCTGCTGCCCAGAGTTTTA3′ R 5′CAGAGGCTACCGAGGACTTG3′ 94°C 30 seconds, 59°C 30 seconds, 72°C 30 seconds, 35 cycles, 72°C 5 minutes, 4°C. *NeuroD2* F 5′GGGACTCGCCTTCTCTCTCT3′R 5′CTATCCCCGAAACTCAGCAG3′ 94°C 30 seconds, 59°C 30 seconds, 72°C 30 seconds, 40 cycles, 72°C 5 minutes, 4°C. *NeuroD3* F 5′CAGTAGTCCCTCGGCTTCAG3′R 5′TAGACTGGGGCAGGAAAGAA3′ 94°C 30 seconds, 59°C 30 seconds, 72°C 30 seconds, 40 cycles, 72°C 5 minutes, 4°C. *Ngn3* F 5′AGGGGACACACGATTAGCAG3′R 5′GGTCTCTTGGGACACTTGGA3′ 94°C 30 seconds, 59°C 30 seconds, 72°C 30 seconds, 38 cycles, 72°C 5 minutes, 4°C. *Notch* F 5′GGTGGACATTGACGAGTGTG3′ R 5′CCCTTGAGGCATAAGCAGAG3′ 94°C 30 seconds, 59°C 30 seconds, 72°C 30 seconds, 27 cycles, 72°C 5 minutes, 4°C. 20 *μ*L of each samples of PCRs were run on 10% TAE PAGE stained with ethidium bromide. Gel image were saved as TIFF files using AlphaImager3300 (Serial no. 981878, Alpha Inc, China.). Gel band quantitation analysis was performed via analysis tools of AlphaImager3300.

## 3. RESULTS

### 3.1. NPCs identification, flt3 expression, and neural progenitor cell growth assay

**Figure 1:**
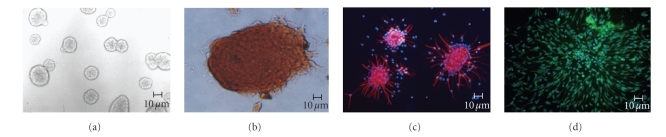
Neural progenitor cell identification and Flt3 expression. (a) Neurospheres under bright field. (b) Flt3-HRP staining on neural spheres. (c) nestin immunofluorescence on neurospheres, RED nestin, BLUE DAPI. (d) Nestin immunofluorescence on single NPC, GREEN nestin, BLUE DAPI.

Primer (P0) cells began to form regular spheres on day 2 after dissection (Figure 1(a)), although some cell death was evident by the presence of cell fragments. More than 98% of the P2 cells were nestin-positive, indicating they were NPCs (Figures 1(c), 1(d)). While more than 95% of cells were positive for FRIL-HRP stain, indicating that most NPCs expressed flt3 protein (Figure 1(b)).

**Figure 2:**
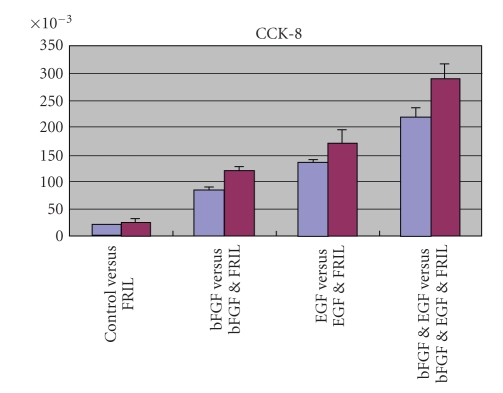
Cell counting assay. Cells were cultured in eight media with different growth factors combination represented in figure by control, FRIL, bFGF, bFGF&FRIL, EGF, EGF&FRIL, bFGF&EGF, bFGF&EGF&FRIL. Cell numbers were transferred into absorption value obtained from microplate reader Bio-Rad Model 550.

Then it came to the NPCs in vitro growth assay. FRIL can promote NPCs growth (*P* < .0001). However, FRIL is less effective than bFGF (*P* < .0001) and EGF (*P* < .0001) (Figure 2).

### 3.2. FRIL delayed formation of neurospheres and induced cell adhesion

**Figure 3:**
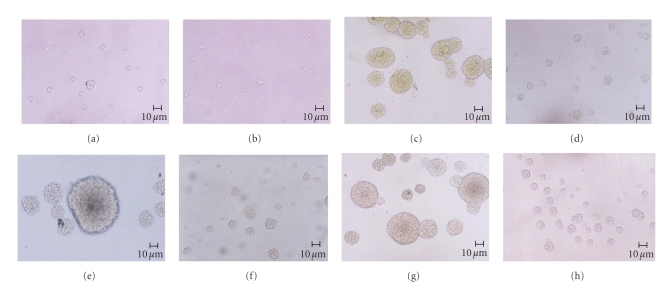
morphological observation of NPCs cultured in different media. (a) NPCs in control medium. (b) NPCs in FRIL medium. (c) NPCs in bFGF medium. (d) NPCs in bFGF& FRIL medium. (e) NPCs in EGF medium. (f) NPCs in EGF&FRIL medium. (g) NPCs in bFGF &EGF medium. (h) NPCs in bFGF&EGF&FRIL medium.

When seeded, NPCs formed spheres later in media with FRIL than those without FRIL. Although there were neurospheres in FRIL containing media, these spheres were smaller than those formed in media without FRIL (Figures 3(b), 3(d), 3(f), 3(h)). Especially in FRIL medium, there were many single/two cells and some small neurospheres which mostly had 3 or 4 cells (Figure 3(b)).

After P3 NPCs were seeded, there was no difference on cell growth in media with/without FRIL from day 1 to day 4 after seeding. However, after day 4, cells in FRIL containing media adhered to the flask surface (Figure 3). And this phenomenon took place again when NPCs were cultured after passage.

NPCs cultured in differentiation media showed difference from day 3 to day 7 after seeding. Cells in serum media seemed to begin to grow on day 3 and covered the bottom of well/flask on day 6/day 7, while cells in FRIL and serum containing media still spoted on the bottom or just formed some colonies. However, from day 7 on cells in FRIL and serum-containing media reached confluency. Most cells showed glial-like morphology. 1∼3 neuron-like cells per well existed in serum-containing media, while 13∼21 cells were present in FRIL and serum-containing media (data not shown).

### 3.3. FRIL reduced GFAP expression while maintained NSE expression

**Figure 4:**
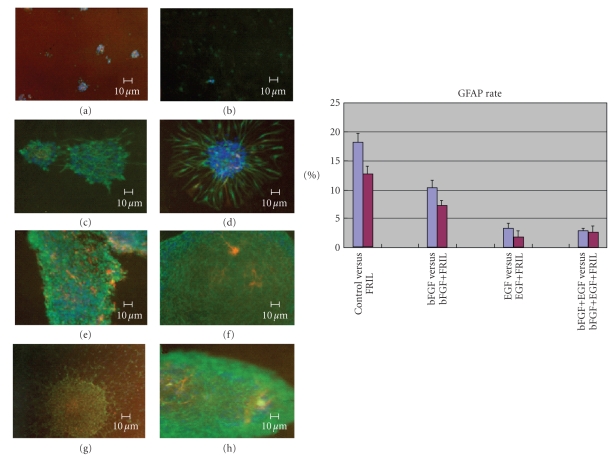
GFAP and nestin immunofluorescence on NPCs cultured in different media. (a) NPCs in control medium. (b) NPCs in FRIL medium. (c) NPCs in bFGF medium. (d) NPCs in bFGF&FRIL medium. (e) NPCs in EGF medium. (f) NPCs in EGF&FRIL medium. (g) NPCs in bFGF&EGF medium. (h) NPCs in bFGF&EGF&FRIL medium. GREEN nestin, RED GFAP, BLUE DAPI.

We immunostained cells, nestin for NPCs, GFAP for astrocytes, NSE/tubulin for neurons, and O4 for oligodendrocytes. Fewer FRIL treated NPCs differentiated into astrocytes than those not treated by FRIL, despite the fact that they had adhered to the plates (Figure 4). Oligodendrocytes were not found. NSE positive cells were not found in culture media containing EGF and EGF&FRIL too. The number of NSE positive cells in FRIL containing media was slightly higher than that without FRIL, although NSE positive rates were small. In addition, tubulin positive cells were not found in any media.

### 3.4. Semiquantitive RT-PCR analysis affirmed FRIL inhibition in glial differentiation

**Figure 5:**
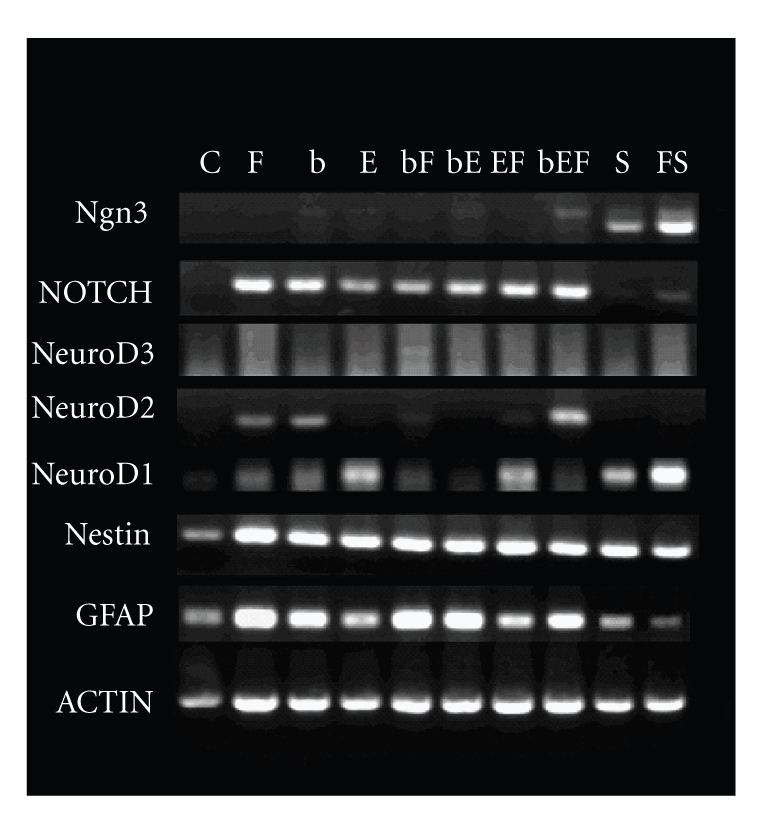
semiquantitive RT-PCR of different genes in NPCs cultured in different media. Lane: (C) NPCs in control medium. (F) NPCs in FRIL medium. (b) NPCs in bFGF medium. (E) NPCs in EGF medium. (bF) NPCs in bFGF&FRIL medium. (bE) NPCs in bFGF&EGF medium. (EF) NPCs in EGF&FRIL medium. (bEF) NPCs in bFGF&EGF&FRIL medium. (S) NPCs in serum medium. (FS) NPCs in serum&FRIL medium. Row: ngn3, neurogenin3. NOTCH, notch. NeuroD3, NeuroD3. NeuroD2, NeuroD2. NeuroD1, NeuroD1. Nestin, nestin. GFAP, GFAP. ACTIN, actin.

On the basis of previous findings [25–27], we chosed 5 genes, namely, *GFAP* and *Notch* (an indicator of GFAP), *NeuroD1*, *NeuroD2*, *NeuroD3*, and *Ngn3* (which serves as upstream regulator of NeuroD) to examine gene expression of cells in eight different cell culture media. *GFAP* and *nestin* were expressed in each group, *nestin* expression was similar, while *GFAP* expression in FRIL containing groups was lower than those without FRIL (Figure 5). *Notch* was not found in control and serum-treated groups, and its expression was quite opposite to that of *GFAP*. Neuronal gene expression was weak, there was no *NeuroD3* expression, *NeuroD2* was only expressed in 3 media, although expression in *NeuroD3* and *Ngn3* was much higher in FRIL and serum media than in serum media. Although FRIL might has no function on neuronal differentiation, these RT-PCR results suggested that FRIL inhibited neural progenitor cells from autodifferentiating into glials.

## 4. DISCUSSION

98.6% nestin positive cells showed that to some extent all cells at P2 were NPCs. 95.7% flt3 expression confirmed the similarities between NPCs and HSCs, although this contradicted to Brazel’s findings in which flt3 was expressed only in NPCs in dorsal root ganglions [12]. The similarities between NPCs and HSCs [28, 29], especially flt3 expression, gave us hints on FRIL-NPCs study.

Previous studies showed that the plant lectin FRIL was successfully extracted from *Dolichos lablab* using a mannose affinity column [11, 15]. Although mannose or *α*-methyl *α*-D-mannoside were reported exerting a negative effects, FRIL had the ability of preserving HSCs in vitro for a long time [21, 22]. In this study, we reported that FRIL could also preserve neural progenitor cells as it does on hematopoietic stem/progenitor cells, it slowed NPC aggregation and growth, on the other hand it inhibited NPC glial autodifferentiation, although it might not affect NPC neuronal autodifferentiation. At present, the common widely studied neural differentiation pathways are Notch, Shh, and so forth [29, 30]. Based on these pathways, both neuronal and glial RT-PCR results are in coincident with the results of immunofluorescence. No matter what kinds of development in which FRIL is involved, the mechanism is still unclear and further researches in signal pathways should be done. Depending on our previous studies that FRIL can affect activities of MAPK, STAT5, and p53, FRIL neural mechanical researches that may be more complicated could be performed.

There have been reports that stem cells can be induced differentiating into neural cells, however cytokines that effectively control these cells are still inadequate. FRIL’s ability to functionally preserve hematopoietic and neural cells may significantly extend the range and time for manipulating these cells. Experiments are underway to find out whether FRIL can improve applications in neural stem cell transplantation, treatment of neural degenerative diseases, and cell therapy, and FRIL’s properties of fuctionally select, preserve, and synchronize hematopoietic cell population could also give some hints.
